# NEK2 promotes the development of ovarian endometriosis and impairs decidualization by phosphorylating FOXO1

**DOI:** 10.1007/s00018-024-05270-8

**Published:** 2024-05-25

**Authors:** Mengxue Wang, Fangyuan Sun, Shucai Zhang, Xiaohui Zhang, Yujun Sun, Ting Yu, Yuanyuan Li, Aifang Jiang, Pengyun Qiao, Chune Ren, Tingting Yang

**Affiliations:** 1 Department of Reproductive Medicine, Affiliated Hospital of Shandong Second Medical University, Weifang, Shandong Province P.R. China; 2 School of Clinical Medicine, Shandong Second Medical University, Weifang, Shandong Province P.R. China; 3 Emergency Department, Affiliated Hospital of Shandong Second Medical University, Weifang, Shandong Province P.R. China; 4 Department of Obstetrics and Gynecology, Zhucheng People’s Hospital, Shandong Second Medical University, Weifang, Shandong Province P.R. China

**Keywords:** NEK2, FOXO1, Decidualization, Endometriosis

## Abstract

Ovarian endometriosis is a common gynecological disease, and one of its most significant symptoms is infertility. In patients with endometriosis, defects in endometrial decidualization lead to impaired endometrial receptivity and embryo implantation, thus affecting early pregnancy and women’s desire to have children. However, the mechanisms underlying the development of endometriosis and its associated defective decidualization are unclear. We find that NEK2 expression is increased in the ectopic and eutopic endometrium of patients with endometriosis. Meanwhile, NEK2 interacts with FOXO1 and phosphorylates FOXO1 at Ser184, inhibiting the stability of the FOXO1 protein. Importantly, NEK2-mediated phosphorylation of FOXO1 at Ser184 promotes cell proliferation, migration, invasion and impairs decidualization. Furthermore, INH1, an inhibitor of NEK2, inhibits the growth of ectopic lesions in mouse models of endometriosis and promotes endometrial decidualization in mouse models of artificially induced decidualization. Taken together, these findings indicate that NEK2 regulates the development of endometriosis and associated disorders of decidualization through the phosphorylation of FOXO1, providing a new therapeutic target for its treatment.

**Supplementary Information:**

The online version contains supplementary material available at 10.1007/s00018-024-05270-8.

## Introduction

Endometriosis is an estrogen-dependent chronic inflammatory disease defined as the presence of endometrial glands and stroma outside the uterine [1]. It affects approximately 190 million women of reproductive age worldwide [2]. It is estimated that 17–44% of patients with endometriosis suffer from ovarian endometriosis [3]. Its most typical symptoms are pelvic pain and infertility [4]. This disease makes a serious impact on the quality of life [5]. According to studies, 25–50% of infertile women also suffer from endometriosis and 30–50% of endometriosis patients experience infertility [6]. Infertility in patients with endometriosis is associated with reduced receptivity of the eutopic endometrium and defective embryo implantation [7]. Although the incidence of endometriosis is still rising, its pathogenesis and the associated mechanisms leading to reduced fertility remain unclear.

Decidualization is essential for endometrial receptivity and embryo implantation [8]. Defect in decidualization of eutopic endometrium is an important factor for infertility in patients with endometriosis [9]. Decidualization refers to the transdifferentiation of endometrial stromal cells surrounding the spiral arteries into large, epithelial-like decidual cells in response to estrogen and progesterone [10]. Endometrial stromal cells have secretory function after decidualization and can secrete a large number of growth factors, among which insulin-like growth factor binding protein-1 (IGFBP1) and prolactin (PRL) are considered to be markers of decidualization [11]. The molecular processes of abnormal gene expression in eutopic endometrium that impair decidualization of endometrial stromal cells in endometriosis patients have been the subject of many studies. It has been shown that the eutopic endometrium of endometriosis-associated infertility patients has lower levels of mitogen inducible gene 6 (Mig-6), and abnormal endometrial decidualization in Mig-6^d/d^ endometriosis mice leads to a significant reduction in the total number of embryos implanted, while simultaneous knockdown of erb-b2 receptor tyrosine kinase 2 (ERBB2) (Mig-6^d/d^ Erbb2^d/d^) reverses the impaired decidualization and reduced fertility caused by Mig-6 [12]. Histone deacetylase 3 (HDAC3) regulates endometrial decidualization and receptivity by targeting collagen type I alpha 1 chain (COL1A1) and collagen type I alpha 2 chain (COL1A2), and knockdown of HDAC3 in mice leads to impaired endometrial decidualization and embryo implantation failure [13]. Because Forkhead box O1 (FOXO1) regulates the transcription of decidualization markers prolactin and IGFBP1, it is considered an indicator of decidualization in endometrial stromal cells [14]. FOXO1 is a representative member of the FOXO family [15]. FOXO1 is involved in oxidative stress [16], cell apoptosis [17], cell autophagy [18], metabolic regulation and immunity [19]. Studies have revealed that patients with endometriosis have a low FOXO1 expression level, and multiple genes affect decidualization of endometrial stromal cells by regulating FOXO1 [20–22]. For example, Notch signaling pathway activity is reduced in decidualized endometrial stromal cells from patients with endometriosis, and this pathway impairs decidualization through downregulation of FOXO1 [21]. Calpain7 promotes nuclear exclusion of FOXO1 by hydrolyzing AKT serine/threonine kinase 1 (AKT1) and inhibits decidualization of human endometrial stromal cells in endometriosis [20]. Post-translational modifications (PTMs), such as phosphorylation [23], ubiquitination [24], acetylation [25] and methylation [26] can regulate the protein function of FOXO1. However, the regulatory mechanisms by which FOXO1 decreases and affects decidualization in endometriosis remain unclear.

Never in Mitosis (NIMA) Related Kinase 2 (NEK2) is a member of the NEKs (Never In Mitosis A (NIMA) related kinases) and is a serine/threonine kinase that exerts biological effects by phosphorylating substrate proteins [27]. Studies have demonstrated that NEK2 is abnormally expressed in a variety of tumors and enhances the proliferation, invasion and drug resistance of tumor cells through multiple signaling pathways [27, 28]. NEK2 promotes the protein stability of programmed death ligand-1 (PD-L1) by phosphorylating PD-L1 at T194/T210 and reduces the efficacy of PD-L1 targeted immunotherapy in pancreatic cancer [29]. In addition, NEK2 stabilizes Yes1 associated transcriptional regulator (YAP1) via phosphorylation at Thr-143, promoting Esophageal Squamous Cell Carcinoma (ESCC) migration and proliferation [30]. NEK2 and NEK2-mediated protein phosphorylation (Ser315 for p53 [31] and Ser352 for growth arrest specific 2 like 1 (GAS2L1) [32]) are also involved in the initiation, progression, metastasis, and adverse prognosis of a range of cancers. The close relationship between NEK2 and tumors makes it an extremely attractive potential therapeutic target, and studies have shown that NEK2 expression is decreased in decidual stromal cells [33]. Hence, it may be related to the disorder of decidualization associated with endometriosis. However, the role of NEK2 in endometriosis and whether it affects the decidualization of the eutopic endometrium in endometriosis have not been investigated.

Therefore, this study aims to investigate the effects of NEK2 overexpression or knockdown on the proliferation, migration, invasion and decidualization of endometrial cells in endometriosis. We investigate whether the NEK2 inhibitor INH1 has therapeutic effects in endometriosis mouse model and artificially induced decidualization mouse model. In addition, we try to study the novel relationship between NEK2 and FOXO1 and the possible mechanisms underlying the regulatory effect of NEK2 on FOXO1 expression in endometrial cells.

## Materials and methods

### Human sample collection and immunohistochemical staining

This study was approved by the Human Investigation Committee of Shandong Second Medical University. All patients provided written, voluntarily informed consent. We recruited 60 patients with ovarian endometriosis and 60 patients without ovarian endometriosis from the Reproductive Medicine Center of the Affiliated Hospital of Shandong Second Medical University. In 60 patients with ovarian endometriosis, 30 patients provided eutopic endometrium and 30 patients provided ectopic endometrium. In 60 patients with non-endometriosis, 30 patients provided normal endometrium in proliferative phase and another 30 patients provided normal endometrium in secretory phase. During surgery, patients with a histological diagnosis of endometriosis and without adenomyosis were divided into the endometriosis group. After surgical examination, patients without signs of endometriosis lesions (tubal infertility and surgery for non-endometriotic ovarian cysts) were divided into the control group. These patients had not received any hormone therapy in the last 6 months. The clinical characteristics of each patient are listed in Supplementary Table S1. For the purposes of the immunohistochemistry tests, the samples were fixed in formalin, embedded in paraffin, and sectioned. The procedure was carried out as previously explained [34]. The intensity of staining was assigned a score of 0 for negative staining, 1 for weak staining, 2 for moderate staining, and 3 for strong positive staining. Positive cell frequency was classified as 0 for 0–5%, 1 for 6-25%, 2 for 26-50%, 3 for 51-75%, and 4 for 76-100%. There were four categories for staining grade: absent (0), weak (1–3), moderate (4–8) or strong (9–12).

### Masson trichrome staning

Tissue sections were routinely deparaffinized into distilled water. Masson staining was then performed with the kit (Solarbio, Shanghai, China) according to the reagent manufacturer’s requirements. Photographs were then taken using a microscope. Collagen fibers were stained blue and muscle fibers were stained red. An increase in blue color indicates a high degree of fibrosis.

### Isolation of primary cells

Primary eutopic endometrial stromal cells (HESC), ectopic endometrial stromal cells (EESC) and normal endometrial stromal cells (ESC) were isolated by the methods described previously [35]. Eutopic and ectopic endometrial tissue from patients with endometriosis and normal endometrial tissue from non-endometriosis patients were cut into 1 mm^3^ fragments. The minced tissue was digested with collagenase type IV (Sigma, St Louis, MO, USA) for 1.5 h at 37 ℃. They were separated by a 76 μm and a 37 μm (pore size) nylon mesh.

### Cell culture

Prof. Sun-wei Guo from Fudan University in Shanghai, China, generously contributed to the endometrial epithelial cell line (11Z), which was established by Anna Strazinski-Powitz. ESC cells are normal endometrial stromal cells. 11Z, ESC, HESC and EESC were cultured in DMEM/F12 medium (HyClone), supplemented with 10% FBS (HyClone), 100 µg/mL penicillin and 100 µg/mL streptomycin. HEK293T cells were grown in DMEM medium (HyClone), supplemented with 10% FBS (HyClone), 100 µg/mL penicillin and 100 µg/mL streptomycin. These cells were grown at 37 °C, in a 5% CO_2_ environment.

### Plasmids and transfection

Human NEK2 and FOXO1 genes were amplified by PCR and then inserted into the pFlag-CMV4 vector and pHA-CMV4 vector. The relevant mutant plasmids were constructed by overlapping PCR techniques. The NEK2 shRNA was produced by oligonucleotide 5′-CCTGTATTGAGTGAGCTGAA-3′. The FOXO1 shRNA was produced by oligonucleotide 5′-GCGGGCTGGAAGAATTCAATT-3′. Transfections were performed using Lipofectamine 2000 (Invitrogen, Shanghai, China) according to the manufacturer’s instructions.

### Induction of HESC decidualization in vitro

HESCs were seeded in six-well plates. When the fusion rate of cells reached 70–80%, the medium was changed to 2% charcoal-stripped phenol red-free medium containing 0.5 mM 8-Br-cAMP (Sigma-Aldrich, St. Louis, MO) and 1 mM medroxyprogesterone acetate (MPA) (Sigma-Aldrich, St. Louis, MO) for 96 h. Every 48 h the medium was changed. The decidualization of HESCs was evaluated by detecting the expression of decidualization-related genes.

### Cell proliferation analysis

The transfected cells were reseeded in 24-well plates (20,000–25,000 cells/plate) and incubated at 37 °C in an incubator. Cell counting was performed for 4 consecutive days.

### Colony formation assay

The reseeded transfected cells (200–800 cells/plate) were then incubated for 10–14 days at 37 °C. The cells were treated with 4% paraformaldehyde for 15 min and stained with crystal violet. Finally, photographs were taken, and the colony number was counted.

### Wound healing assay

The transfected cells were reseeded in 6-well plates, the next day, when 100% confluence was achieved, the cells were scratched with a 200 µl pipette tip, washed with PBS and photographed. Photographs were taken again after 24 h of incubation.

### Transwell migration assay and Matrigel invasion assay

Two hundred microliters of serum-free DMEM/F12 medium (CORNING) were mixed with transfected cells (1 × 10^5^) and added to the upper chamber. Then, 600 µL of medium containing 10% FBS was added to the lower chamber. To conduct invasion tests, 40 µl of Matrigel gel (BD Biosciences, Bedford, MA, USA) was diluted with serum-free DMEM/F12 at a concentration of 1:8, added to the upper chamber, and incubated for 1 h at 37 °C. After 12–24 h, the cells and matrix gel were removed from the upper chamber. The cells were then stained with crystal violet for 10 min after being fixed with 4% paraformaldehyde for 15 min. Pictures were taken, and the number of cells was counted.

### RNA isolation, cDNA synthesis, and real-time PCR

After total RNA was extracted by TRIzol kit (Takara), the cDNA was produced by reverse transcription using the PrimeScript RT reagent kit (Takara). The expression levels of mRNA were measured using SYBR Green PCR Master Mix (Takara) and CFX96 real-time PCR detection system (Bio-Rad). The sequences of primers used are listed in Supplementary Table S2.

### Western blot

Cells were lysed using lysis buffer (Beyotime, Shanghai, China, P0013) and subsequently centrifuged at 12,000 rpm for 10 min at 4 °C. After adding 5× loading buffer, the supernatant was heated at 100 °C for 10 min. SDS-PAGE was used to separate the proteins, which were then transferred to PVDF membranes. The membranes were washed three times with TBST. After being blocked for 1 h at room temperature with TBST solution containing 5% non-fat milk, the membranes were incubated with the primary antibody overnight at 4 °C. The fluorescent secondary antibody was applied to the membranes, which were then incubated at room temperature for 1 h. The proteins were visualized by an Odyssey instrument. The procedure was carried out as previously explained [36]. Antibodies used for Western blot were summarized in Supplementary Table S3.

### Immunofluorescent analysis

On cell crawlers, cells (1.2 × 10^5^) were seeded and then incubated for 24 h. The cells were treated with 0.05% Triton X-100 for 10 min after being fixed in 4% paraformaldehyde for 10 min. The 1 h was spent blocking the cells with BSA. The cells were incubated with primary antibody at 4 °C overnight. The cells were incubated with a fluorescent-conjugated secondary antibody for 1 h at room temperature and stained with DAPI. Finally, the cells were observed and photographed under a fluorescence microscope (ZEISS).

### Immunoprecipitation

The lysate was added to the collected cells and then incubated for 30 min on ice. Then, the mixture was centrifuge for 10 min at 12,000 rpm at 4 °C. 5% of the supernatant was used as the input, the antibodies and protein A/G beads were added, and the mixture was then incubated at 4 °C overnight. Normal mouse or rabbit IgG was used as a negative control. The beads were washed five times with lysate, then loading buffer was added and boiled for 10 min before Western blot.

### Ubiquitin detection

Cells were transfected with the plasmids HA-NEK2, Flag-FOXO1 (WT, S184A, S184D) and HA-Ubi plasmids, and MG132 (100 µmol/L) was added for 8 h before collection. Then, co-IP and Western blot experiments were carried out.

### Animal experiments

The Animal Experimentation Ethical Committee of Shandong Second Medical University approved all mice experimental procedures. For our research, we selected 6-week-old female C57BL/6 mice.

Endometriosis mouse model. Donor mice (*n* = 7) were injected intramuscularly with estradiol benzoate (100 µg/kg) in the thighs once every two days. After one week, the recipient mice were given intraperitoneal injections of the donor mouse uterus, which was divided into 1 mm^3^ pieces and thoroughly mixed. After establishing the endometriosis model for 1 week, the mice in the experimental group (*n* = 7) were injected intraperitoneally with the NEK2 inhibitor INH1 (100 mg/kg), and the mice in the control group were injected intraperitoneally with the same amount of solvent every 3 days. Endometriosis lesions were collected and measured after the mice were killed after one month. The samples were fixed, embedded, and sectioned, and subjected to hematoxylin-eosin (H&E) staining and immunohistochemistry.

Artificially induced in vivo decidualization. The endometriosis model of C57BL/6 mice was first established, as described above. After 1 month, the mice were anesthetized, their ovaries were removed bilaterally, and after 2 weeks, 100 ng of estradiol was injected for 3 consecutive days. After two days of rest, the mice were injected with 1 mg of progesterone and 10 ng of estradiol for 3 consecutive days. Six hours after the last hormone injection, the mice were anaesthetized, and 20 µL of sesame oil was injected into one uterine horn to artificially induce decidualization, while the other uterine horn was left untreated as a control. Then, 1 mg of progesterone and 10 ng of estradiol were injected for 5 consecutive days. During this process, the mice were treated with the NEK2 inhibitor INH1 or solvent, for the time shown in supplementary Fig. 5C. Six hours after the last injection, the mice were sacrificed, and the wet weights of both uterine horns were recorded for each mouse. Uterine tissues were collected and fixed in 4% paraformaldehyde for histological and immunohistochemical analysis.

### Statistical analysis

All experiments were independently repeated at least three times. All the statistical analyses were performed using GraphPad Prism 9.0 software. The statistical analyses were presented as mean ± SEM. Data analysis methods included Mann–Whitney test, Student’s t-test and one-way ANOVA. The relationship between the two variables was analyzed using Spearman’s correlation analysis. P values < 0.05 were considered significant, and ns was not significant.

## Results

### NEK2 expression is increased in the endometrium of patients with endometriosis and negatively correlated with FOXO1

**Fig. 1:**
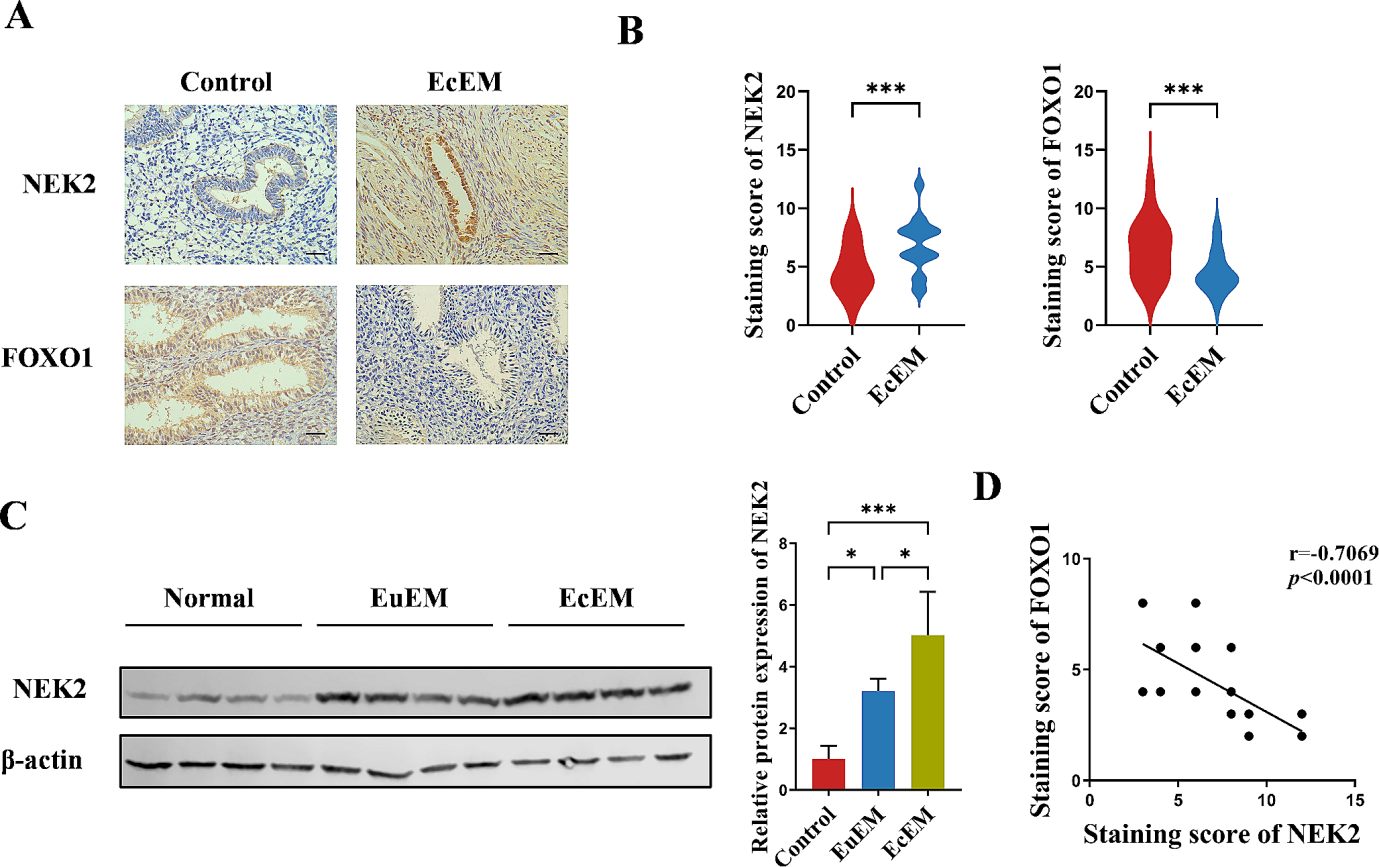
NEK2 expression is increased in endometriosis tissues and negatively correlated with FOXO1. **A** The expression of NEK2 and FOXO1 in normal proliferative endometrium and ectopic endometrium were examined by immunohistochemistry (scale bar, 20 μm). **B** Semiquantitative immunohistochemical analysis of normal and ectopic tissues for NEK2 and FOXO1 (*n* = 30). **C** Western blot was used to detect the expression of NEK2 protein in endometrium (*n* = 4). **D** Spearman’s correlation analysis was performed to determine correlation between NEK2 and FOXO1 expression in ectopic tissues (*n* = 30)(All data represent mean ± SEM. **P < 0.01, ***P < 0.001, ****P < 0.0001)

To explore the expression of NEK2 in endometriosis, the ectopic endometrium from patients with endometriosis was subjected to immunohistochemical experiments, and the normal proliferative endometrium served as a control. IHC revealed significantly elevated levels of NEK2 expression in the ectopic endometrium of patients with endometriosis compared with controls (Fig. 1A, B). Western blot data further confirmed the increased levels of NEK2 in the ectopic endometrium of patients with endometriosis (Fig. 1C). Interestingly, FOXO1 was reduced in endometriosis (Fig. 1A, B). Our findings demonstrated that NEK2 and FOXO1 were negatively correlated in endometriosis (Fig. 1D).

### NEK2 promotes the progression of endometriosis in vitro and in vivo

**Fig. 2:**
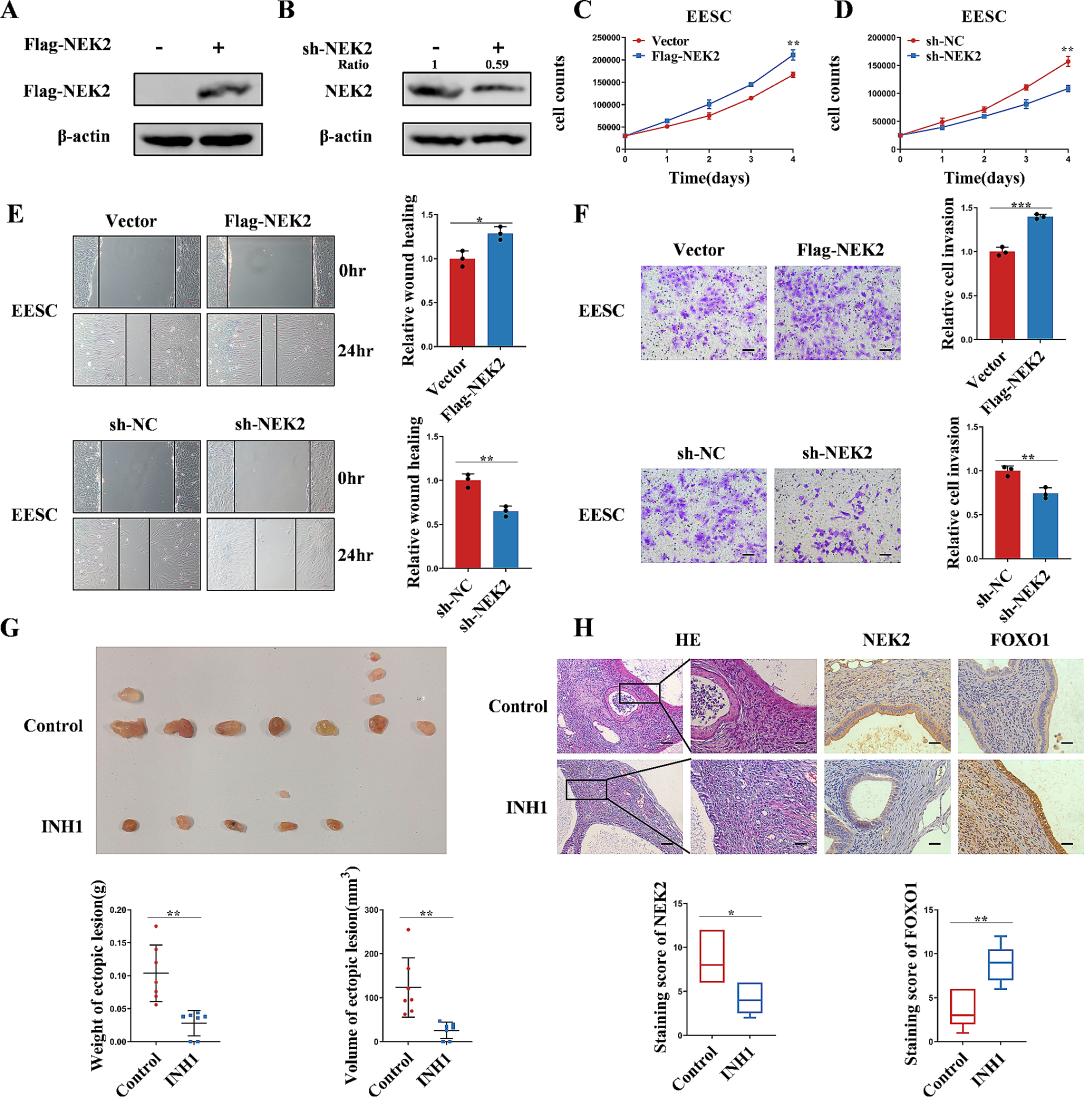
NEK2 promotes cell proliferation, migration and invasion in vitro and in vivo. **A, B** The levels of NEK2 overexpression and knockdown in EESC cells were analyzed by Western blot. **C, D** NEK2 was overexpressed or knocked down in EESC cells, and then cell proliferation assay was performed. **E** NEK2 was overexpressed or knocked down in EESC cells, and then wound healing assay was performed. **F** NEK2 was overexpressed or knocked down in EESC cells, and then matrigel invasion assay was performed. **G** The volume and weight of endometriosis lesions in the control and experimental groups. **H** Hematoxylin-eosin staining of the endometriosis lesions in the control and INH1 groups (200× and 400×). Immunohistochemical analysis was performed to detect the expression of NEK2 and FOXO1 in endometriosis lesions in the control and experimental groups (400×, scale bar, 20 μm). (All data represent mean ± SEM. *P < 0.05, **P < 0.01, ***P < 0.001, ****P < 0.0001)

To determine whether NEK2 is involved in the development of endometriosis, we overexpressed and knocked down NEK2 in EESC and 11Z cells, respectively. We verified the successfully overexpression and knockdown down of NEK2 by western blot (Fig. 2A, B and Supplementary Fig. 1A, B). We discovered that overexpression of NEK2 enhanced the proliferation of endometriotic cells (Fig. 2C and Supplementary Fig. 1C, E, F). In contrast, knockdown of NEK2 inhibited cell proliferation (Fig. 2D and Supplementary Fig. 1D-F). In addition, we performed wound healing, transwell migration and invasion experiments, and the results showed that NEK2 promoted the migration and invasion ability of EESC and 11Z cells (Fig. 2E, F and Supplementary Fig. 1G-J). To further demonstrate whether NEK2 has a promoting effect on endometriosis. We treated the cells with INH1, an inhibitor of NEK2. First, we assayed the IC50 of INH1 in 11Z and EESC cells (Supplementary Fig. 2A). The above experiments revealed that INH1 inhibited the proliferation, migration and invasion of endometriosis cells (Supplementary Fig. 2B-F). We also overexpressed NEK2 in normal endometrial stromal cells and then performed cell proliferation, wound healing, transwell migration and invasion assays. The results showed that overexpression of NEK2 promoted the proliferation, migration and invasion of ESC cells (Supplementary Fig. 3A-D). These findings suggest that NEK2 promotes the proliferation, migration and invasion of endometriosis cells.

Studies have shown that INH1 decreases the protein level of NEK2 and inhibits cancer cell growth in vivo and in vitro [37]. In this study, we used C57BL/6 female mice to establish endometriosis models to determine how NEK2 affects the growth of endometriosis in vivo (Supplementary Fig. 4A). The results demonstrated that the weight and volume of endometriotic lesions in the experimental mice were significantly lower than those in the control group (Fig. 2G and Supplementary Fig. 4B). Immunohistochemical staining for E-cadherin, vimentin, and Masson’s trichrome staining of sections of ectopic lesions from a mouse model of endometriosis proved that the tissue used was endometriosis (Supplementary Fig. 4C). We performed hematoxylin-eosin (H&E) staining and immunohistochemical analysis of lesions. We found that the expression levels of NEK2 were reduced and the expression levels of FOXO1 were increased after INH1 treatment (Fig. 2H). In summary, NEK2 promotes the progression of endometriosis both in vitro and in vivo.

### NEK2 impairs decidualization in vitro and in vivo

Defective decidualization of eutopic endometrium is an important factor for infertility in patients with endometriosis [9]. Therefore, we hypothesized that NEK2 impairs the decidualization of endometrial stromal cells. To verify our hypothesis, we performed immunohistochemical analysis of eutopic endometrium from patients with endometriosis and used normal secretory phase endometrium as a control. NEK2 levels were increased and FOXO1 levels were decreased in eutopic endometrium as compared with normal controls (Supplementary Fig. 5A, B).

**Fig. 3:**
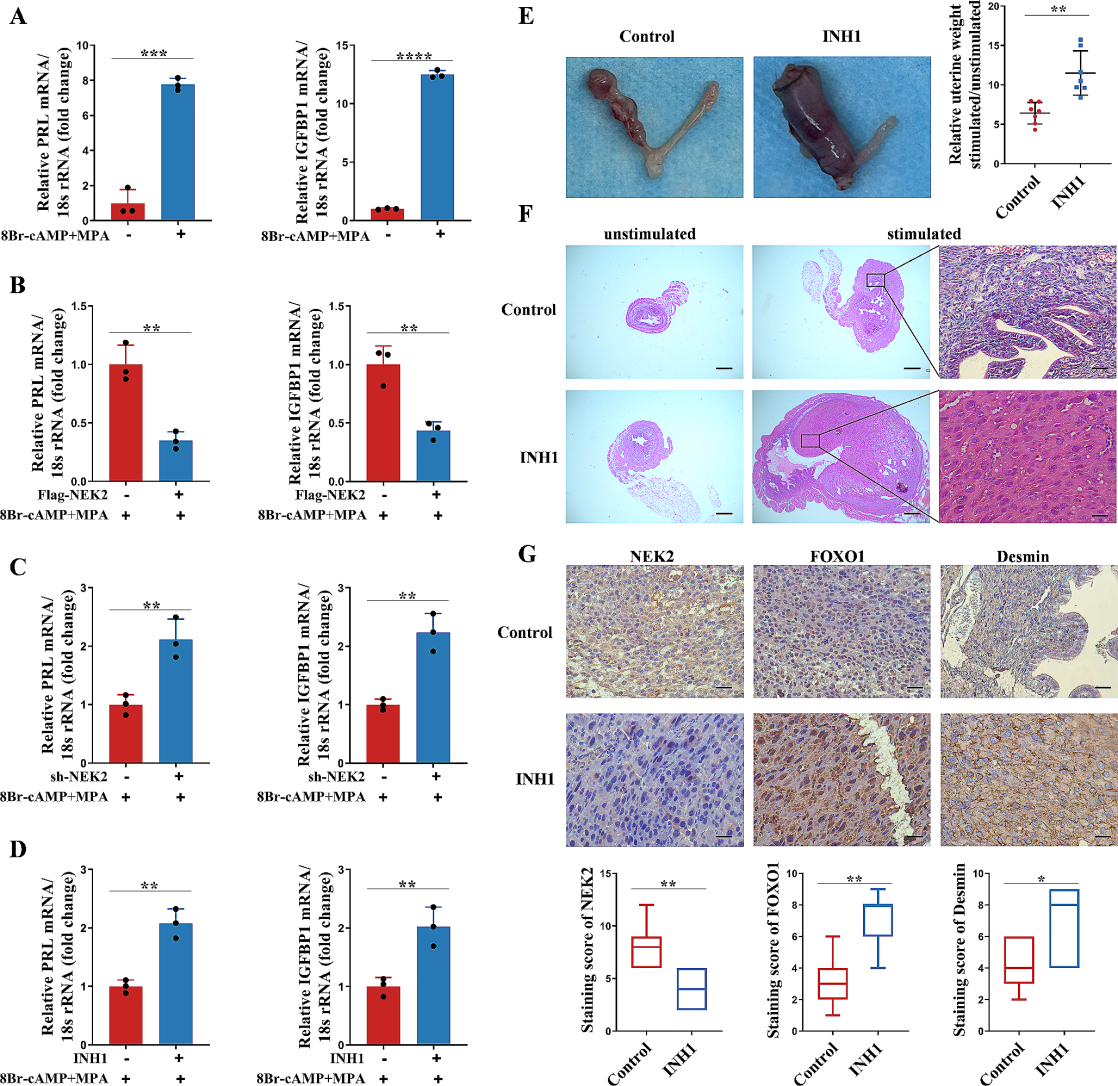
NEK2 damages decidualization of endometriotic endometrial stromal cells in vitro and in vivo. **A** Total RNA was isolated from HESC treated with or without 8-Br-cAMP and MPA. The mRNA levels of PRL and IGFBP1, markers of decidualization, were measured by RT-qPCR in HESC. **B** NEK2 was overexpressed in HESC and then treated with 8-Br-cAMP and MPA for 4 days. The mRNA levels of decidualization markers PRL and IGFBP1 were detected in HESC by RT-qPCR. **C** NEK2 was knocked down in HESC and then treated with 8-Br-cAMP and MPA for 4 days. The mRNA levels of decidualization markers PRL and IGFBP1 were detected in HESC by RT-qPCR. **D** HESC were treated with INH1, followed by treatment with 8-Br-cAMP and MPA for 4 days. The mRNA levels of decidualization markers PRL and IGFBP1 were detected in HESC by RT-qPCR. **E** General morphology of the unstimulated or stimulated uterine side and the ratio of the weight of the stimulated to the weight of the unstimulated uterus in the control and INH1 groups with endometriosis after artificially induced decidualization. **F** Hematoxylin-eosin staining of unstimulated and stimulated uteri in the control and INH1 groups (scale bar, 20 μm). **G** Immunohistochemistry was used to detect the expression of NEK2, FOXO1 and Desmin in the stromal cells of the stimulated uterus in control and INH1 groups (scale bar, 20 μm). (All data represent mean ± SEM. A-D: *n* = 3; E-G: *n* = 7. *P < 0.05, **P < 0.01, ***P < 0.001, ****P < 0.0001)

To further study whether NEK2 is involved in decidualization, we performed an in vitro decidualization assay using HESCs. We treated HESCs with 8 Br-cAMP + MPA for 4 days followed by RT-qPCR and found increased mRNA levels of decidualization markers PRL and IGFBP1, suggesting that we successfully induced decidualization in vitro (Fig. 3A). We then examined the impact of NEK2 on decidualization by altering the expression of NEK2 in HESCs. First, we transfected vector or Flag-tag-NEK2 plasmids into HESCs and then treated HESCs with 8 Br-cAMP + MPA for 4 days. These results showed that overexpression of NEK2 impaired decidualization of HESC cells compared to controls (Fig. 3B). In contrast, transfection of shRNA-NEK2 in HESC cells, or treatment of cells with INH1, decreased the levels of NEK2 in HESC cells and promoted decidualization (Fig. 3C-D). Taken together, these findings indicate that NEK2 impairs decidualization of endometrial stromal cells in vitro.

To verify whether NEK2 damages decidualization of endometrial stromal cells in vivo, we first established a mouse model of endometriosis. After one month, we induced decidualization of the uterine horn in mice. The mice in the experimental group were intraperitoneally injected with NEK2 inhibitor INH1, and the mice in the control group were intraperitoneally injected with the same volume of solvent. The wet weight of the uterus was then measured, and it was found that the stimulated/control horn weight ratio in the experimental group was substantially higher than that in the control group (Fig. 3E). In addition, histological analysis of the uterus in the control and INH1 groups revealed decidual cells morphology, with an increase in the size of the stimulated horn in the experimental group compared to the control group (Fig. 3F). Finally, we performed immunohistochemical analysis of the stimulated uterus and found that the expression levels of NEK2 were decreased in endometrial stromal cells but the expression levels of FOXO1 were increased after INH1 treatment. Desmin, a marker of decidualization, also increased in the experimental group (Fig. 3G). These data reveal that NEK2 impairs decidualization in vitro and in vivo.

#### NEK2 interacts with FOXO1 and phosphorylates FOXO1 at Ser184

**Fig. 4:**
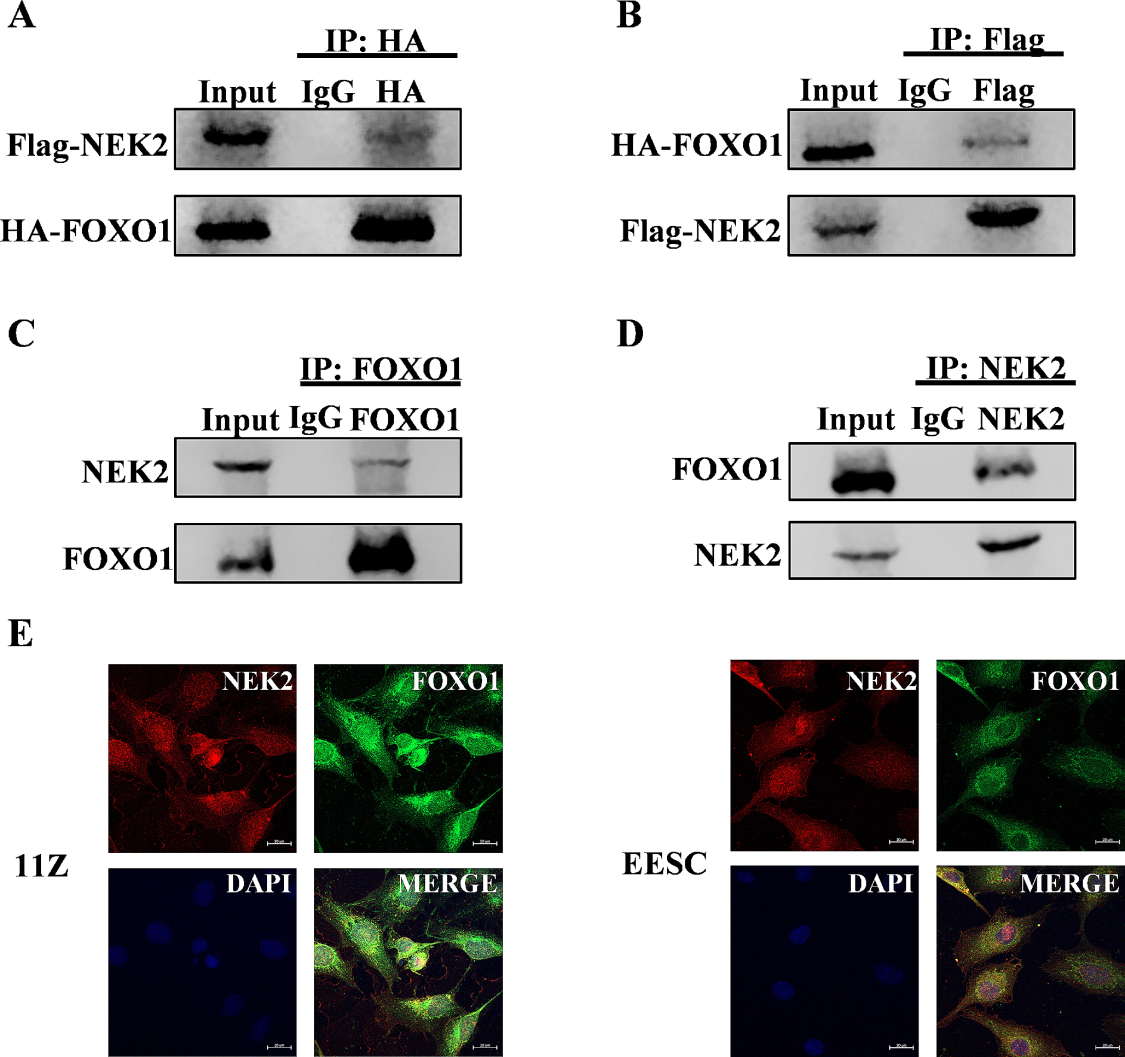
NEK2 interacts with FOXO1. **A, B** Flag-tagged NEK2 and HA-tagged FOXO1 were transfected into HEK293T cells. The interaction between them was verified by immunoprecipitation assay and western blot. **C, D** The interaction of endogenous NEK2 and FOXO1 proteins in EESC cells was verified by immunoprecipitation and western blot. **E** The analysis of the localization of NEK2 (red) and FOXO1 (green) in endometriosis cells by confocal immunofluorescence microscopy

In the previous experiments, we demonstrated that NEK2 is negatively correlated with FOXO1, and to further explore the regulatory mechanisms involved, we conducted a series of experiments. Co-IP revealed that NEK2 and FOXO1 interact in 293T and EESC cells (Fig. 4A-D). We then used immunofluorescence to examine the distribution zones of NEK2 and FOXO1 in 11Z and EESC cells (Fig. 4E). These data suggest that FOXO1 is a novel binding protein for NEK2.

**Fig. 5:**
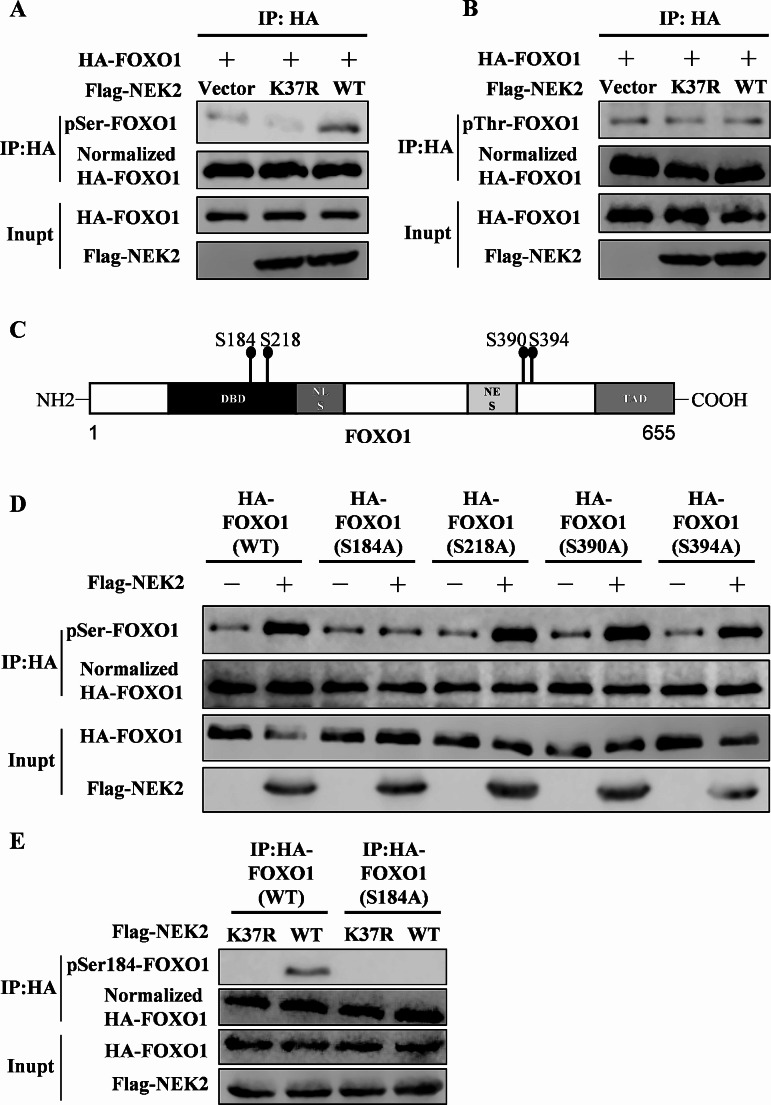
NEK2 phosphorylates FOXO1 at Ser184. **A, B** HA-FOXO1 and Flag-NEK2 (empty vector or K37R or WT) were transfected into HEK293T cells. Anti-HA antibody was used to immunoprecipitate cell lysates. FOXO1 phosphorylation and protein levels were analyzed by Western blot with indicated antibodies. **C** Potential NEK2 phosphorylation sites in the FOXO1 protein sequence. **D** HEK293T cells were co-transfected with HA-tagged FOXO1(WT or mutants) and Flag-tagged NEK2 (vector or WT) plasmids. Immunoprecipitation was performed with anti-HA antibodies, followed by Western blot analysis using the specified antibodies. **E** HEK293T cells were co-transfected with HA-FOXO1 (WT or S184A) and Flag-NEK2 (WT or K37R) plasmids. Immunoprecipitation was performed with anti-HA antibodies, followed by Western blot analysis using the specified antibodies

It was previously shown that NEK2 can function by phosphorylating its downstream substrates [27]. To determine whether NEK2 phosphorylates FOXO1, we overexpressed Flag-tagged NEK2 (WT or kinase-inactive (K37R)) and HA-tagged FOXO1 in 293T cells. Compared with the control vector or kinase inactive NEK2, WT NEK2 increased the serine phosphorylation levels of FOXO1 (Fig. 5A), but NEK2 had no effect on threonine phosphorylation levels (Fig. 5B). Then we used the GPS 5.0 algorithm to predict four potential phosphorylation sites for NEK2 in the FOXO1 protein sequence (Fig. 5C). To clarify the specific site of action, we performed point mutations on the potential sites of FOXO1. Mutated S184A of FOXO1 abrogated the effect on serine phosphorylation levels (Fig. 5D). In addition, we produced an antibody that recognizes the phosphorylation of Ser184 on FOXO1. We discovered that NEK2 had no discernible impact on the phosphorylation of the mutant FOXO1 (S184A) (Fig. 5E). In conclusion, NEK2 can phosphorylate FOXO1 at Ser184.

### NEK2 downregulates the protein stability of FOXO1

**Fig. 6:**
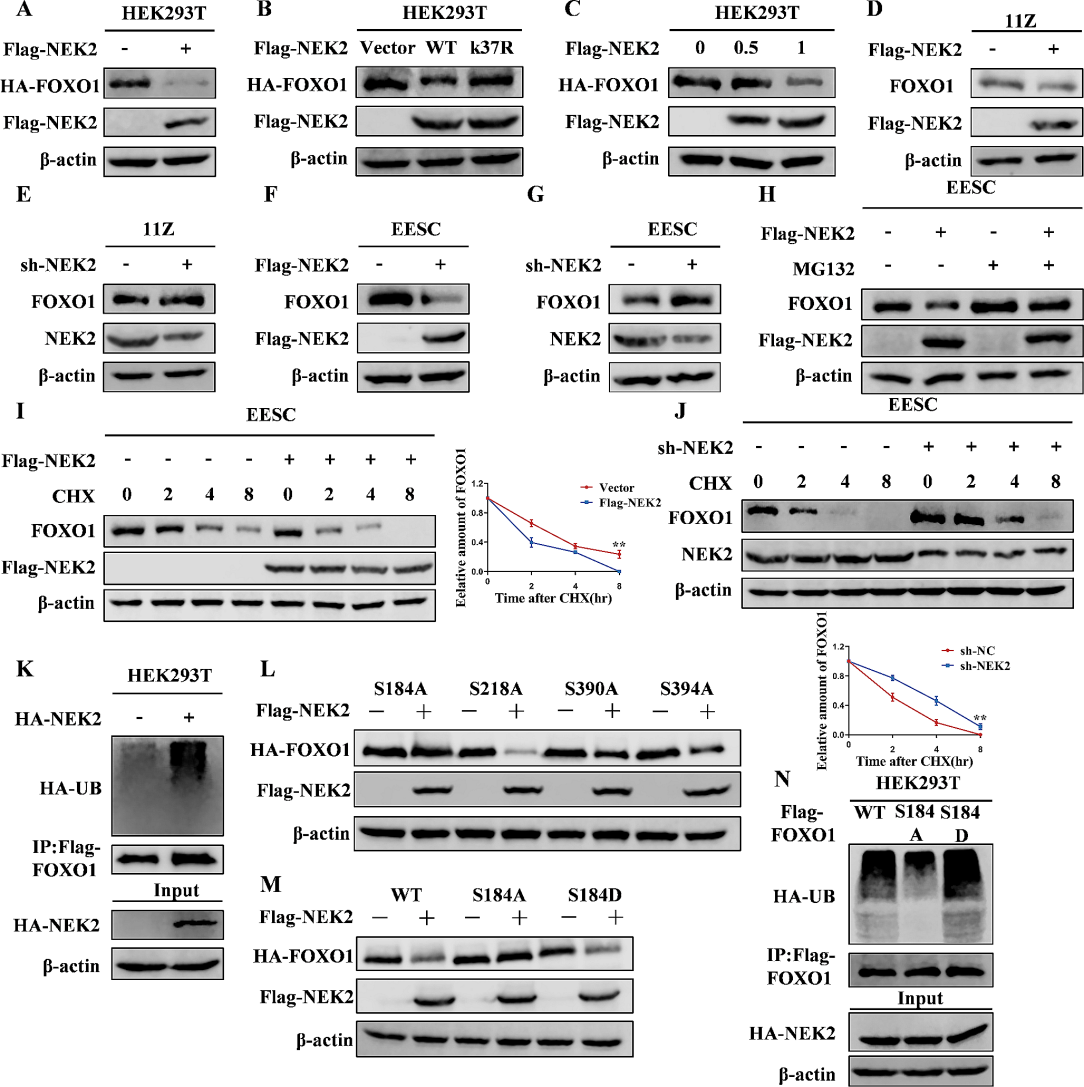
NEK2 inhibits the protein stability of FOXO1. **A** NEK2 and FOXO1 were overexpressed in HEK293T cells, followed by Western blot. **B** The vector, Flag-tagged NEK2 (WT or K37R) and HA-tagged FOXO1 were transfected in HEK293T cells. The expression of NEK2 and FOXO1 were detected by Western blot. **C** The Flag-tagged NEK2 (0, 0.5, 1 µg) and HA-tagged FOXO1 were transfected in HEK293T cells. The expression of NEK2 and FOXO1 were detected by Western blot. **D-G** Overexpression or knockdown of NEK2 was performed in 11Z and EESC cells. Western blot was used to detect the expression of NEK2 and FOXO1. **H** EESC cells were transfected with Flag-NEK2 and then treated with MG132 (100 µmol/L) for 8 h. Western blot was performed. **I, J** NEK2 was overexpressed or knocked down in EESC cells and then treated with CHX for the indicated times. Western blot analysis was performed. **K** The Flag-tagged FOXO1 and HA-tagged NEK2 were transfected in HEK293T cells. 48 h after transfection, the cells were treated with MG132 for 8 h, followed by IP and Western blot assays. **L** The Flag-tagged NEK2 and HA-tagged FOXO1 mutants were transfected in HEK293T cells. The expression levels of FOXO1 mutants were detected by Western blot. **M** The Flag-tagged NEK2 and HA-tagged FOXO1 (WT, S184A or S184D) were transfected in HEK293T cells. The expression levels of FOXO1 were detected by western blot. **N** The HA-tagged NEK2 and Flag-tagged FOXO1 (WT, S184A or S184D) were transfected in HEK293T cells. After 48 h of transfection, the cells were treated with MG132 for 8 h before IP and Western blot. (All data represent mean ± SEM. *P < 0.05, **P < 0.01, ***P < 0.001, ****P < 0.0001)

To investigate the effect of NEK2 on FOXO1 protein stability, we transfected flag-tagged NEK2 and HA-tagged FOXO1 into 293T cells. Then, we performed western blot, and the results revealed that NEK2 decreased FOXO1 protein stability (Fig. 6A). To investigate whether NEK2-regulated FOXO1 protein stability is dependent on its kinase activity. We transfected vector, Flag-NEK2 (WT or K37R) in 293T cells. The results showed that the protein levels of FOXO1 were not altered by vector and Flag-NEK2 (K37R) compared with Flag-NEK2 (WT) (Fig. 6B). We subsequently found that NEK2 decreased FOXO1 protein levels in a dose-dependent manner (Fig. 6C). To measure the levels of endogenous FOXO1 protein, we overexpressed or knocked down NEK2 in endometriosis cells. The endogenous FOXO1 protein levels were decreased when 11Z and EESC cells were transfected with Flag-NEK2. In contrast, we knocked down NEK2 in 11Z and EESC cells and detected elevated endogenous FOXO1 protein levels (Fig. 6D-G).

To determine whether NEK2 affects FOXO1 stability through the proteasome pathway, we overexpressed NEK2 in EESC cells and treated the cells with MG132 (100 µmol/L). The outcomes demonstrated that MG132 reversed the FOXO1 downregulation caused by NEK2 (Fig. 6H). In addition, we exposed EESC cells to cycloheximide (CHX) to examine the impact of NEK2 on the protein half-life of FOXO1. As anticipated, NEK2 shortened the half-life of the FOXO1 (Fig. 6I, J). Next, we determined whether NEK2 could regulate the ubiquitination levels of FOXO1. We found that overexpression of NEK2 in HEK293T cells altered the levels of ubiquitination of FOXO1 (Fig. 6K). We next aimed to verify whether NEK2 affects protein stability by phosphorylating FOXO1. Mutant plasmids of FOXO1 or Flag-NEK2 plasmids were transfected in 293T cells. The stability of HA-FOXO1 (S184A) was found to be unchanged (Fig. 6L, M). Subsequently, we transfected HA-NEK2 and Flag-FOXO1 (WT, S184A, S184D) into HEK293T cells and performed ubiquitination experiments. Interestingly, decreased and increased ubiquitination of FOXO1 were observed in the FOXO1 S184A and FOXO1 S184D mutants, respectively (Fig. 6N). Taken together, these data suggest that NEK2 promotes the degradation of FOXO1 via the ubiquitin proteasome pathway.

### FOXO1 Ser184 mediates cell proliferation, migration, invasion and decidualization of endometrial stromal cells

**Fig. 7:**
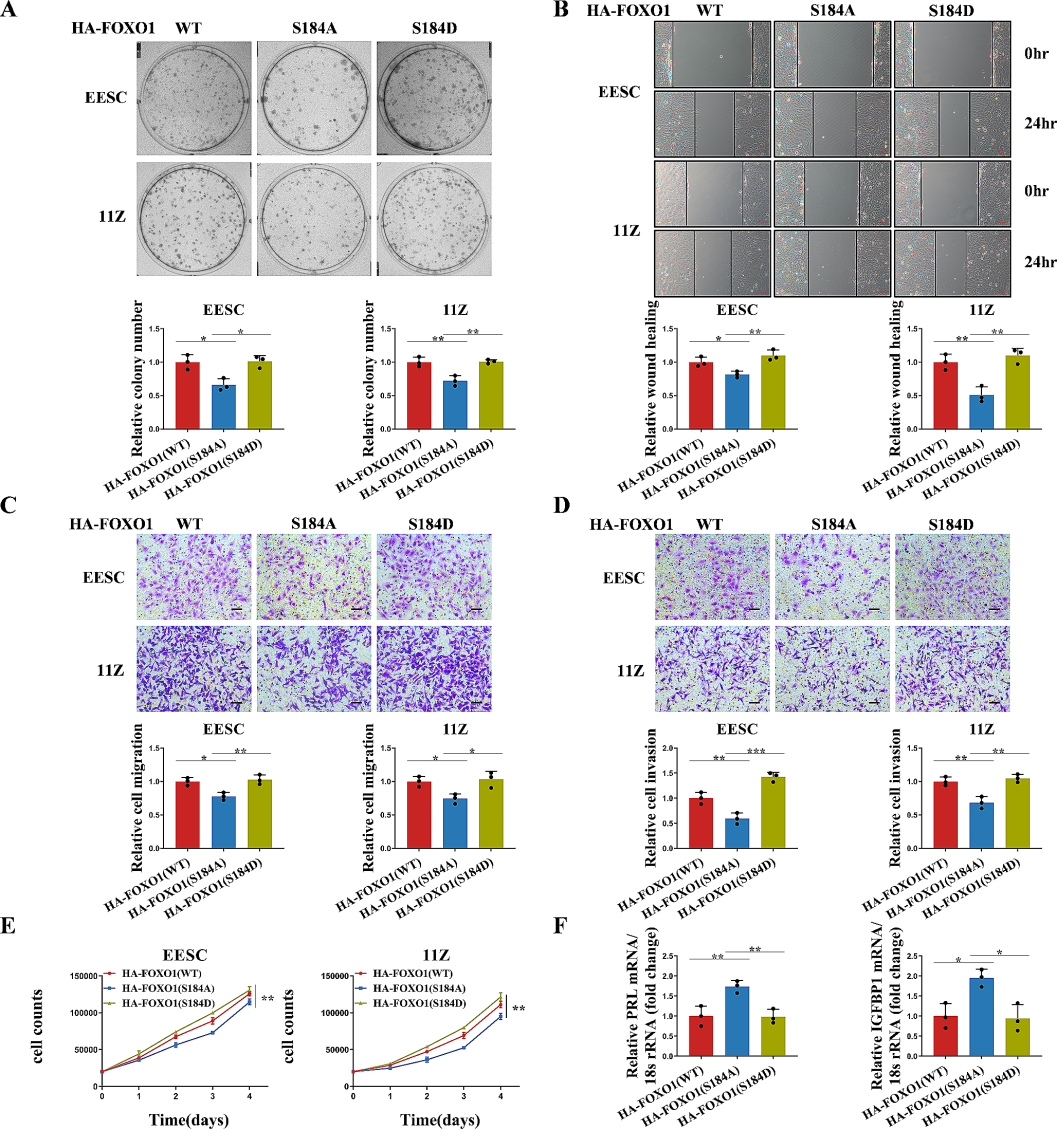
FOXO1 Ser184 phosphorylation promotes cell proliferation, migration, invasion and impairs decidualization. **A** FOXO1 was knocked down with sh-FOXO1, and then HA-FOXO1 (WT, S184A or S184D) was transfected in EESC and 11Z cells. Then clone formation assay was performed. **B** FOXO1 was knocked down with sh-FOXO1, and then HA-FOXO1 (WT, S184A or S184D) was transfected in EESC and 11Z cells. Then wound healing assay was performed. **C, D** FOXO1 was knocked down with sh-FOXO1, and then HA-FOXO1 (WT, S184A or S184D) was transfected in EESC and 11Z cells. Then transwell migration assay and matrigel invasion assay were performed. **E** FOXO1 was knocked down with sh-FOXO1, and then HA-FOXO1 (WT, S184A or S184D) was transfected in EESC and 11Z cells. Then cell proliferation assay was performed. **F** FOXO1 was knocked down with sh-FOXO1, and then HA-FOXO1 (WT, S184A or S184D) was transfected in HESC cells and then treated with 8-Br-cAMP and MPA for 4 days. The mRNA levels of decidualization markers PRL and IGFBP1 were detected in HESC by RT-qPCR. (All data represent mean ± SEM. *n* = 3 *P < 0.05, **P < 0.01, ***P < 0.001, ****P < 0.0001)

To verify the effect of the FOXO1 Ser184 on the biological significance of endometriosis cells, we conducted a rescue experiment. The cell proliferation capacity of the mutant FOXO1 (S184A) was reduced compared to the control groups (Fig. 7A, E). As shown in Fig. 7B-D, the mutant FOXO1 (S184D) significantly increased the ability of cell migration and invasion. In HESC cells, we discovered that expression of the mutant FOXO1 (S184A) increased the mRNA levels of the decidualization markers PRL and IGFBP1 compared to FOXO1 expression (WT or S184D) (Fig. 7F). In conclusion, FOXO1 Ser184 phosphorylation promotes cell proliferation, migration, invasion and impairs decidualization of endometrial stromal cells.

### NEK2 regulates cell proliferation, migration, invasion and decidualization through FOXO1

**Fig. 8:**
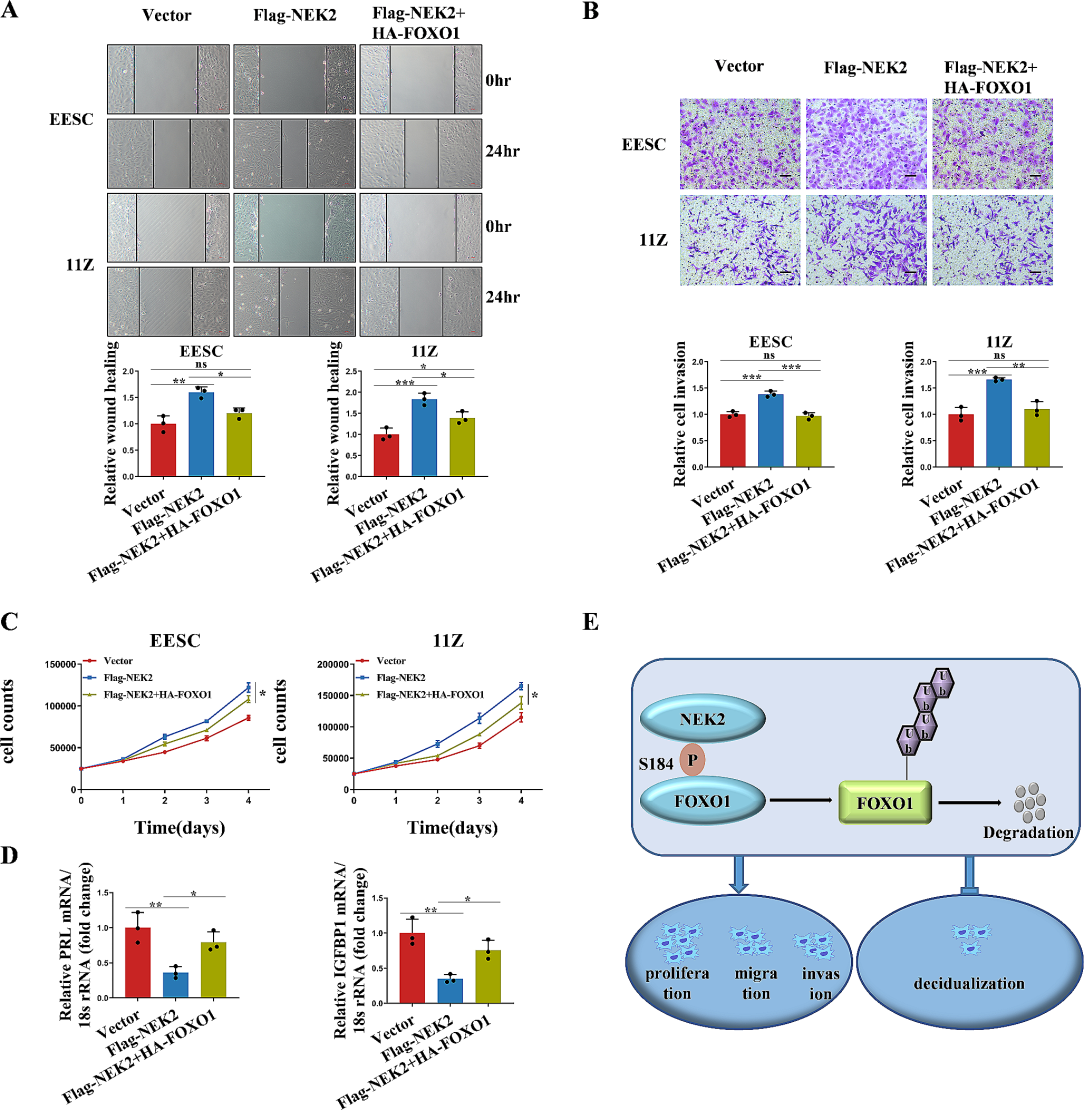
NEK2 regulates cell proliferation, migration, invasion and decidualization through FOXO1. **A** The EESC and 11Z cells were transfected with vector, Flag-NEK2 and Flag-NEK2 + HA-FOXO1, respectively. Wound healing assay was performed. **B** The EESC and 11Z cells were transfected with vector, Flag-NEK2 and Flag-NEK2 + HA-FOXO1, respectively. Matrigel invasion assay was performed. **C** The EESC and 11Z cells were transfected with vector, Flag-NEK2 and Flag-NEK2 + HA-FOXO1, respectively. Cell proliferation assay was performed. **D** The HESC cells were transfected with vector, Flag-NEK2 and Flag-NEK2 + HA-FOXO1, respectively. Then treated with 8-Br-cAMP and MPA for 4 days. The mRNA levels of decidualization markers PRL and IGFBP1 were detected by RT-qPCR. **E** A working model in which NEK2 regulates endometriosis progression and decidualization through phosphorylating the FOXO1 Ser184 site. (All data represent mean ± SEM. *n* = 3 *P < 0.05, **P < 0.01, ***P < 0.001, ****P < 0.0001)

Because NEK2 interacts with FOXO1, we hypothesized that FOXO1 is necessary for NEK2 to regulate cell proliferation, migration, invasion, and decidualization. We first transfected flag-tagged NEK2 or HA-tagged FOXO1 into EESC and 11Z cells. Figure 8C and Supplementary Fig. 6A demonstrated that the overexpression of NEK2 promoted cell proliferation, but restoration of FOXO1 expression attenuated this effect. We also found that restoration of FOXO1 expression attenuated the promoting effect of NEK2 by using wound healing, transwell migration and matrigel invasion experiments (Fig. 8A, B and Supplementary Fig. 6B). Subsequently, we transfected flag-tagged NEK2 or HA-tagged FOXO1 into HESC cells, and then treated with 8 Br-cAMP + MPA for 4 days. The results showed that the mRNA levels of the decidualization markers PRL and IGFBP1 decreased after NEK2 overexpression and increased after restoration of FOXO1 expression (Fig. 8D). These findings suggest that FOXO1 is indeed required for NEK2 to regulate cell proliferation, migration, invasion and decidualization.

## Discussion

This study revealed the increase of NEK2 in endometriosis compared to controls, and NEK2 was negatively correlated with FOXO1. NEK2 promoted the proliferation, migration, and invasion of endometriotic cells and impaired the decidualization of endometriosis eutopic endometrial stromal cells by phosphorylating FOXO1 at Ser184 and reducing its stability. These results were also verified in animal experiments.

Endometriosis is usually considered a benign disease, but it has many similar features to cancer, such as migration and invasion [38]. Many malignancies, including cervical cancer [39], hepatocellular carcinoma [40], gastric cancer [41] and lung cancer [42], have been reported to abnormally express NEK2. Therefore, we examined the expression of NEK2 in normal and ectopic endometrium by immunohistochemistry. The NEK2 protein levels were found to be significantly increased in endometriosis. In addition, we investigated the role of NEK2 in endometriosis by altering NEK2 expression in endometriosis cells. The outcomes demonstrated that NEK2 promoted cell proliferation, migration and invasion. In addition, NEK2 inhibitor INH1 inhibited cell proliferation, migration, invasion and suppressed the growth of endometriosis lesions in mouse models of endometriosis. These findings suggest that NEK2 promoted the development of endometriosis.

Infertility is one of the most significant symptoms of endometriosis, and it is much more common in patients with endometriosis than in the general population [43]. It has been shown that defective decidual response and embryo implantation failure occur in animal models of endometriosis [12; 44]. Decreased decidual response in HESC cells from patients with endometriosis [45]. In addition, it has been shown that the levels of NEK2 are reduced in decidual endometrial stromal cells [33]. In our study, immunohistochemical results showed that NEK2 expression was significantly increased in the secretory phase eutopic endometrium of endometriosis than in the normal secretory phase endometrium. Decidualization of endometrial stromal cells can be induced in vitro [46]. By altering the levels of NEK2 in HESC cells and inducing their decidualization, we found that NEK2 decreased the mRNA levels of the decidualization markers PRL and IGFBP1 and impaired the decidualization of HESC cells. We then further studied the impact of NEK2 on decidualization using an artificially induced decidualization mouse model. INH1 is an inhibitor of NEK2, and we found that INH1 increased decidualization in artificially stimulated decidualization mice compared with controls. These data suggest that NEK2 impaired decidualization in the endometrium.

FOXO1 has a significant impact on cell proliferation, migration, invasion [47], and decidualization [48]. When cells are in different biological environments, the corresponding protein post-translational modifications act on FOXO1 protein, affecting the expression of downstream target genes by altering its transcriptional activity or subcellular localization, and ultimately affecting the biological behaviors of cells [49]. Aberrant activation of cyclin dependent kinase 1 (CDK1) promote cell proliferation and survival through phosphorylation and inhibition of FOXO1, thereby promoting tumorigenesis [50]. Dual specificity tyrosine phosphorylation regulated kinase 1 A (DYRK1A) promoted the development of B-cell acute lymphoblastic leukemia through the phosphorylation of FOXO1 [23]. In endometriosis FOXO1 was phosphorylated by the phosphatidylinositol 3 kinase (PI3K)/protein kinase B (AKT) signaling pathway and degraded by ubiquitination after exiting the nucleus, resulting in decreased expression of the downstream decidualization marker IGFBP1 [22]. We performed prediction by GPS 5.0 algorithm and found four potential NEK2 phosphorylation sites in the FOXO1 protein sequence. Our results suggested that NEK2 interacted with FOXO1 and phosphorylated FOXO1 at Ser184. In addition, we examined the effect of NEK2 phosphorylation of FOXO1 on its stability, and interestingly, FOXO1 protein stability was reduced. Then we focused on the function of NEK2 in regulating proliferation, migration, invasion and decidualization by targeting FOXO1 protein phosphorylation. The results determined that FOXO1 Ser184 phosphorylation promoted cell proliferation, migration, invasion and impaired decidualization. We performed dependent experiments and found that restoration of FOXO1 expression reversed the NEK2-induced increase in proliferation, migration, invasion, and defective decidualization.

In conclusion, our experimental data show that NEK2 is increased in both ectopic and eutopic endometrium from women with ovarian endometriosis. The evidence from cellular experiments suggests that NEK2 directly interacts with FOXO1 and phosphorylates at the Ser184 site inhibits the protein stability of FOXO1, thereby enhancing proliferation, migration, invasion and impairing decidualization of stromal cells in endometriosis (Fig. 8E). Results consistent with cellular experiments are obtained in mouse models of endometriosis and in artificially induced decidualization models. Our study lays a theoretical foundation for developing new strategies for the treatment of endometriosis and its associated endometrial decidualization defects by targeting NEK2 and FOXO1.

### Electronic supplementary material

Below is the link to the electronic supplementary material.

Supplementary Material 1

## Data Availability

Enquiries about data availability should be directed to the authors.
