# Supplementary material for: NEK2 promotes the development of ovarian endometriosis and impairs decidualization by phosphorylating FOXO1
Source: Cell Mol Life Sci. 2024 May 25;81(1):237. doi: 10.1007/s00018-024-05270-8 (PMC11127904; doi:10.1007/s00018-024-05270-8)
Supplement: Supplementary file 1 — Supplementary Material 1 [file 18_2024_5270_MOESM1_ESM.docx]

**List of Supplementary Data**

**
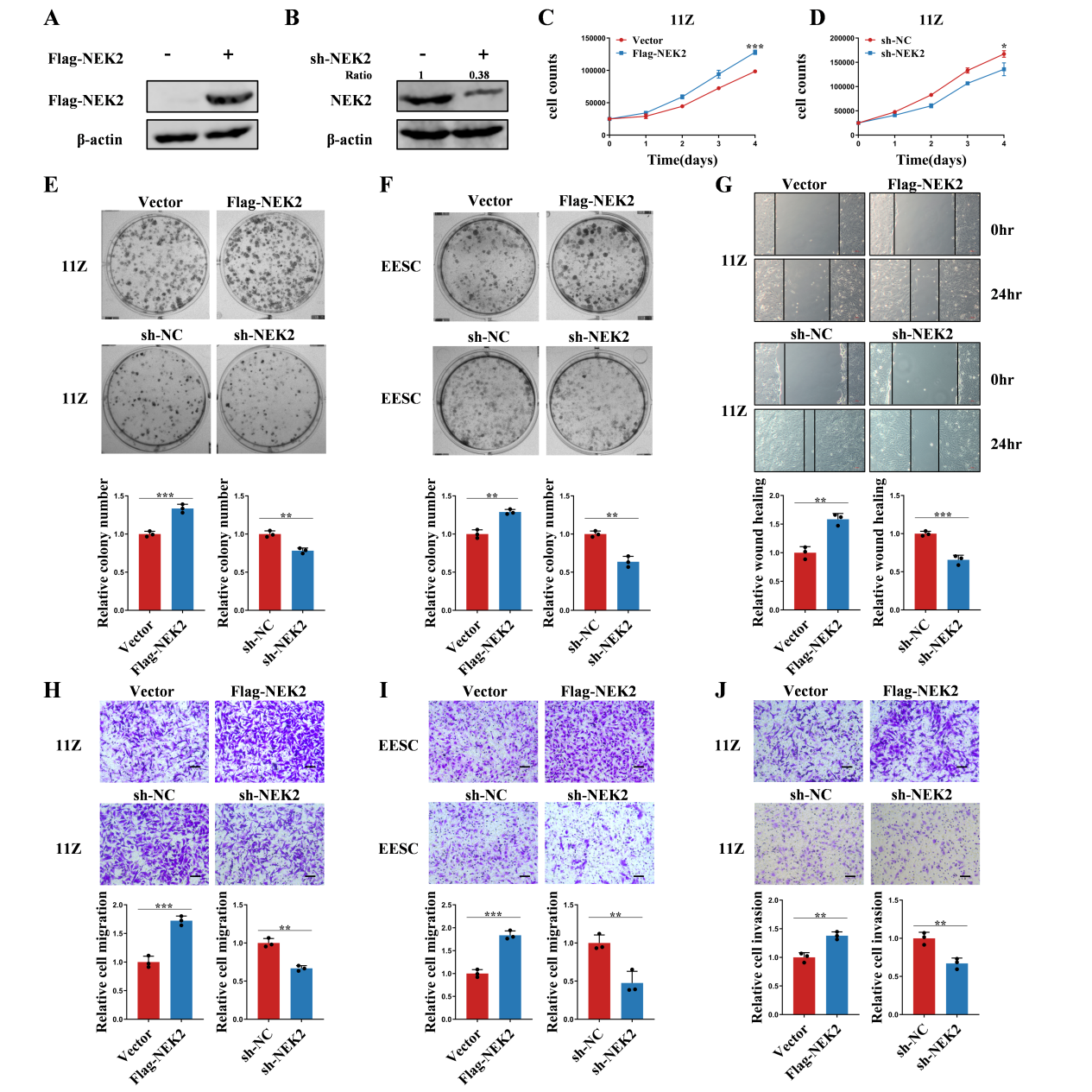
**

**Supplementary fig 1. NEK2 promotes cell proliferation, migration and invasion in vitro.**

**A, B** Western blot analysis revealed overexpression and knockdown levels of NEK2 in 11Z cells.

**C, D** NEK2 was overexpression or knocked down in 11Z cells, then cell proliferation assay was performed.

**E, F** NEK2 was overexpression or knocked down in 11Z and EESC cells, then the clone formation assay was performed.

**G** NEK2 was overexpression or knocked down in 11Z cells, and then wound healing assay was performed.

**H, I** NEK2 was overexpression or knocked down in 11Z and EESC cells, and then transwell migration assay was performed.

**J** NEK2 was overexpression or knocked down in 11Z cells, and then matrigel invasion assay was performed.

(All data represent mean ± SEM. *P<0.05, **P<0.01, ***P<0.001)


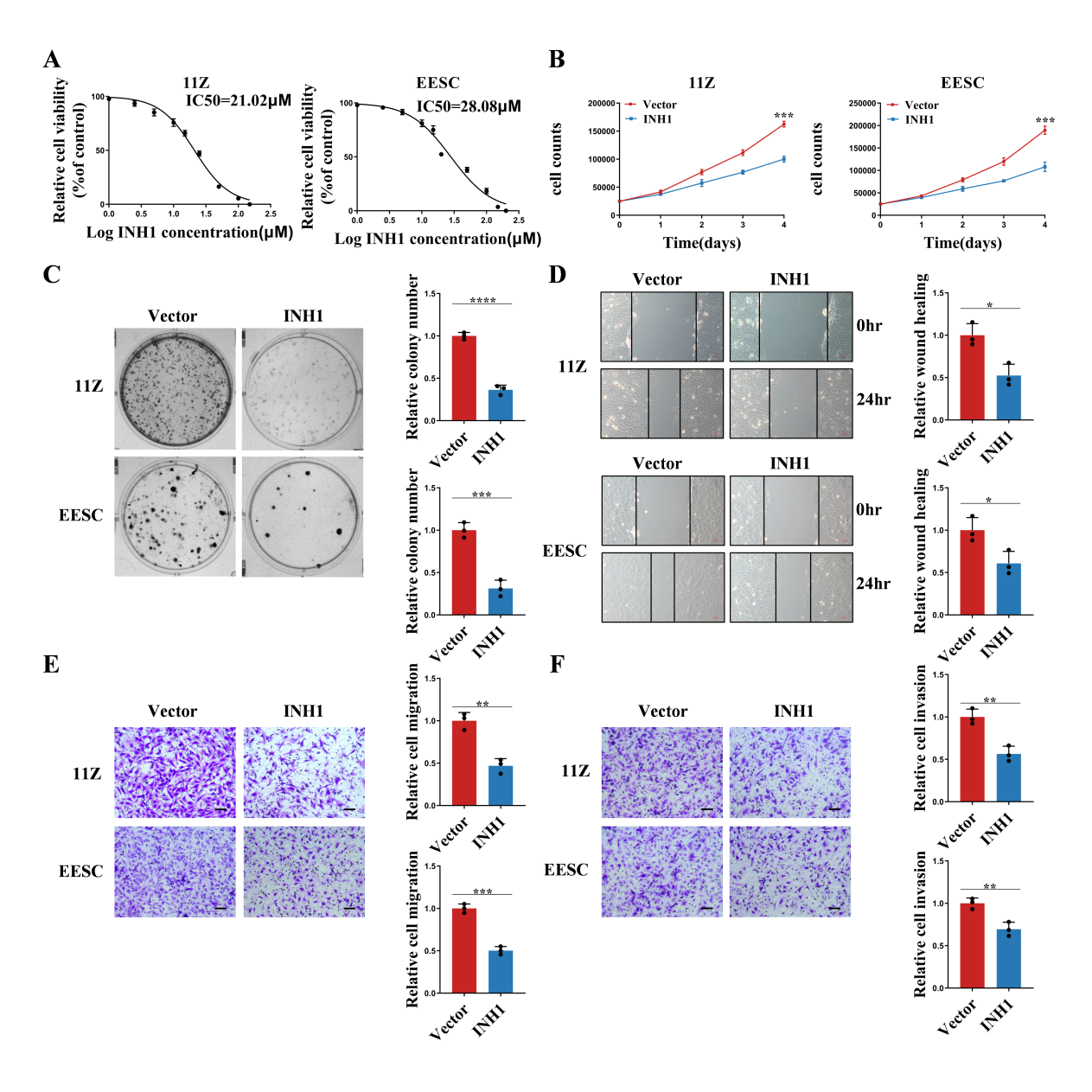


**Supplementary fig 2. NEK2 inhibitor INH1 inhibits cell proliferation, migration and invasion.**

**A** 11Z and EESC cells were treated with different concentrations of INH1 and their IC50 was determined.

**B** The 11Z and EESC cells were treated with INH1. Cell proliferation assay was performed.

**C** The 11Z and EESC cells were treated with INH1. Clone formation assay was performed.

**D** The 11Z and EESC cells were treated with INH1. Wound healing assay was performed.

**E, F** The 11Z and EESC cells were treated with INH1. Transwell migration assay and matrigel invasion assay were performed.

(All data represent mean ± SEM. *P<0.05, **P<0.01, ***P<0.001, ****P<0.0001)


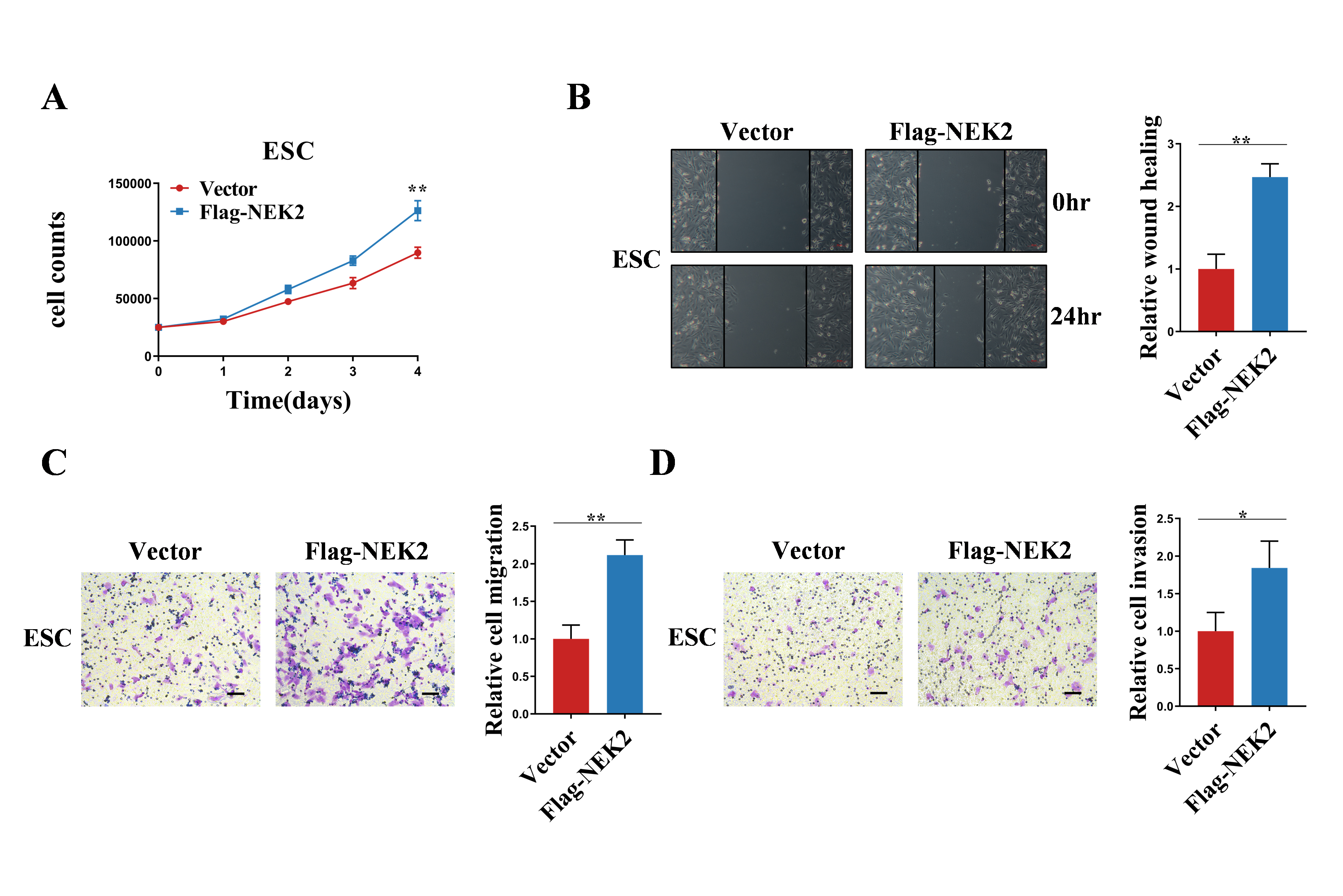
**Supplementary fig 3. NEK2 promotes ESC cells proliferation, migration and invasion.**

**A** NEK2 was overexpression in ESC cells, then cell proliferation assay was performed.

**B** NEK2 was overexpression in ESC cells, and then wound healing assay was performed.

**C** NEK2 was overexpression in ESC cells, and then transwell migration assay was performed.

**D** NEK2 was overexpression in ESC cells, and then matrigel invasion assay was performed.

(All data represent mean ± SEM. *P<0.05, **P<0.01)


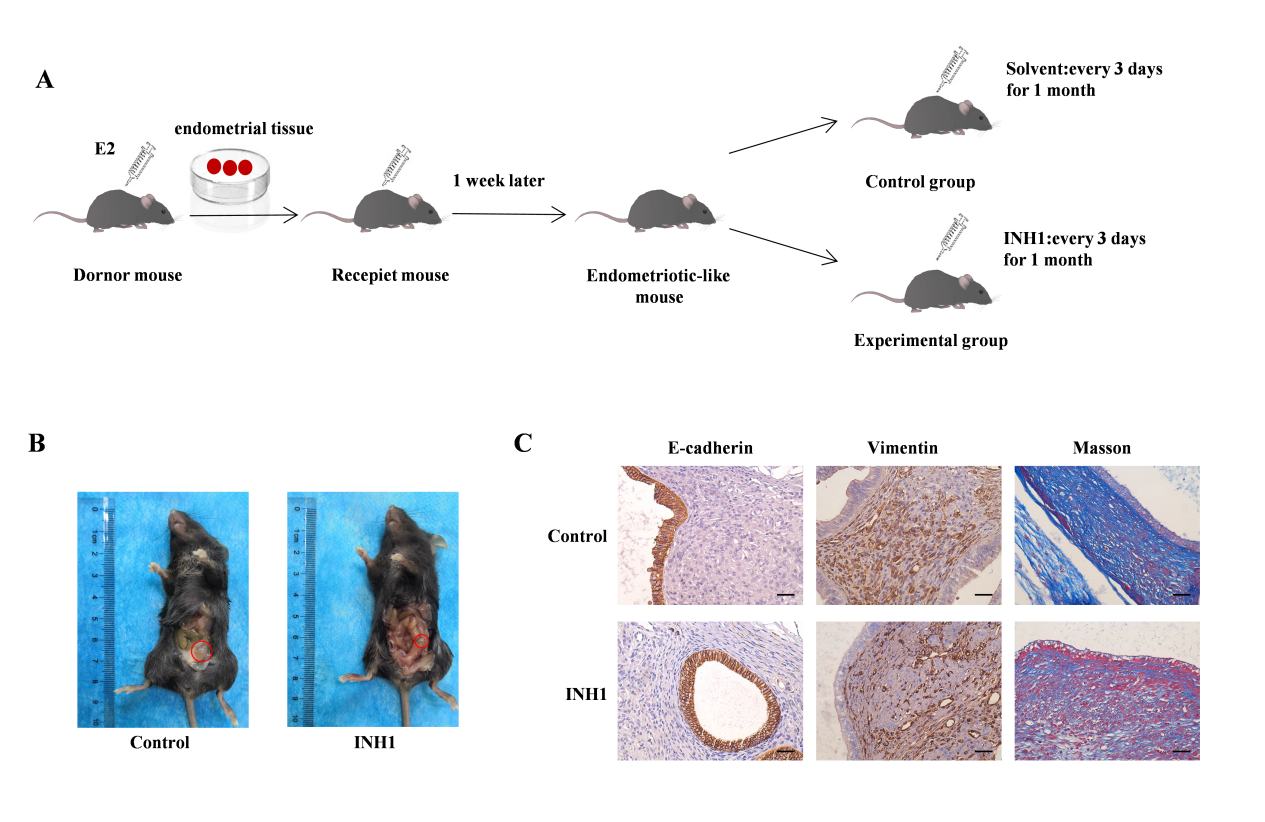


**Supplementary fig 4. NEK2 promotes the progression of endometriosis in vivo.**

**A** Schematic diagram of the establishment of a mouse model of endometriosis.

**B** Representative images of the endometriosis lesion. Endometriosis lesions are circled in red.

**C** IHC stains on control and INH1 lesions. E-cadherin stain for epithelial cells, Vimentin stain for stromal cells. Masson Trichrome for fibrosis (scale bar, 20 µm).

**
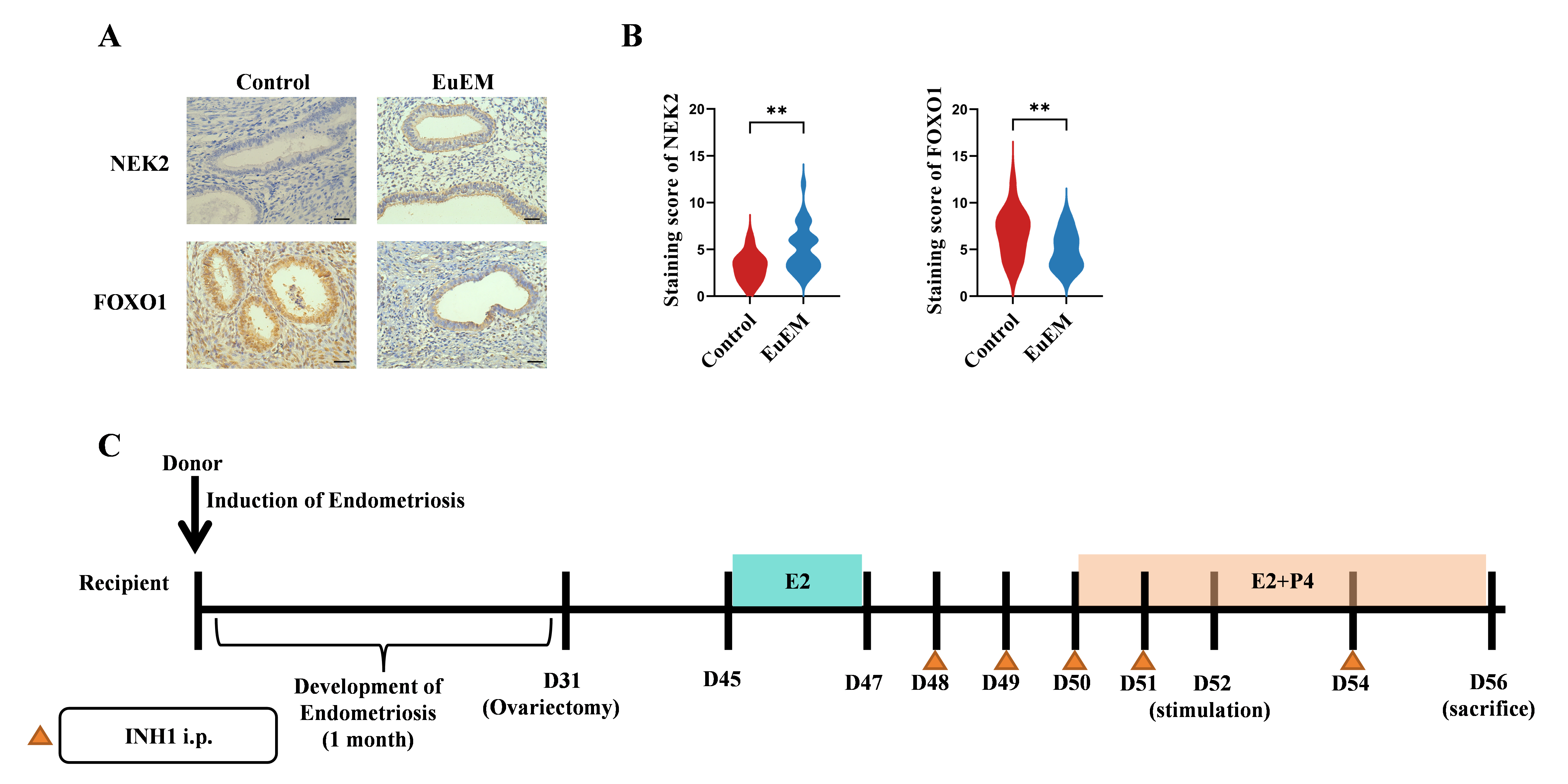
**

**Supplementary fig 5. NEK2 is highly expressed in eutopic endometrium of endometriosis**

**A** The expression of NEK2 and FOXO1 in normal secretory endometrium and eutopic endometrium were examined by immunohistochemistry (scale bar, 20 µm).

**B** Semi-quantitative immunohistochemical analysis of NEK2 and FOXO1 was performed in normal and eutopic tissues (n=30).

**C** Experimental design to study the effect of NEK2 on decidualization of endometriosis.

(All data represent mean ± SEM. **P<0.01)


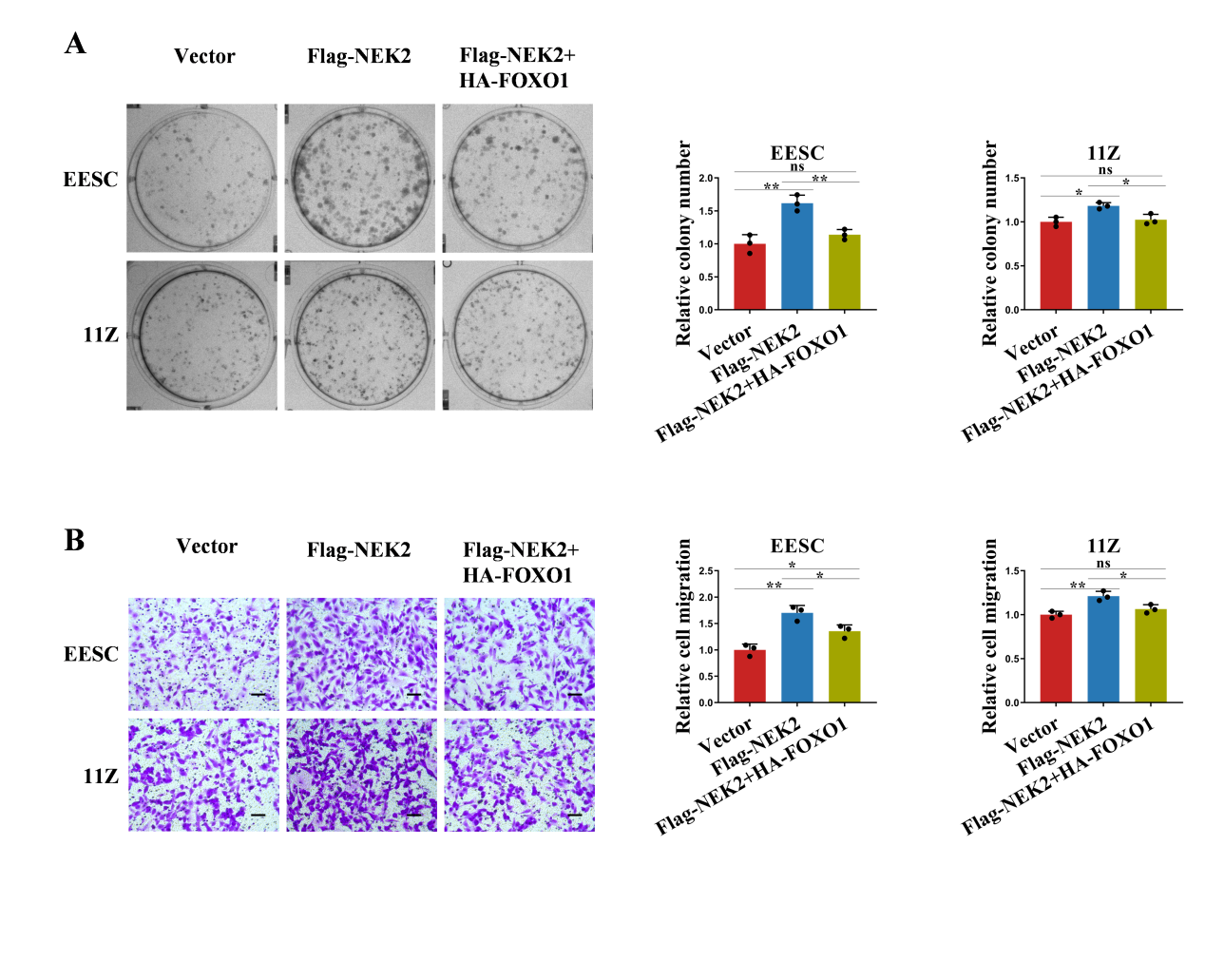


**Supplementary fig 6. NEK2 regulates cell proliferation and migration through FOXO1.**

**A** The EESC and 11Z cells were transfected with vector, Flag-NEK2 and Flag-NEK2+HA-FOXO1, respectively. The clone formation assay was performed.

**B** The EESC and 11Z cells were transfected with vector, Flag-NEK2 and Flag-NEK2+HA-FOXO1, respectively. Transwell migration assay was performed.

(All data represent mean ± SEM. *P<0.05, **P<0.01)

**Supplementary Table 1. Clinical characteristics of patients.**

|  | Control | | endometriosis | |
| --- | --- | --- | --- | --- |
|  | Normal endometrium | | eutopic endometrium | ectopic endometrium |
| Mensteual cycle phase | Proliferative | secretory | secretory | Proliferative |
| No of case | 30 | 30 | 30 | 30 |
| Age (mean ± SD) | 33.1±4.2 | 31.8±2.1 | 32.7±4.9 | 33.3±3.6 |
| rASRM stag  Ⅲ  Ⅳ | -  - | -  - | 12  18 | 14  16 |

**Supplementary Table 2. The primers used for real-time PCR in this study.**

| **Gene** | **Sense (5’-3’)** | **Anti-sense (5’-3’)** |
| --- | --- | --- |
| NEK2 | CGGAAGTTCCTGTCTCTGGCA | TTCAGGTCCTTGCACTTGGACT |
| FOXO1 | TGGACATGCTCAGCAGACATC | TTGGGTCAGGCGGTTCA |
| PRL | CATCAACAGCTGCCACACTT | CGTTTGGTTTGCTCCTCAAT |
| IGFBP1 | CTATGATGGCTCGAAGGCTC | TTCTTGTTGCAGTTTGGCAG |
| 18S | GTTGAACCCCATTCGTGATG | GCCTCACTAAACCATCCAA |

**Supplementary Table 3. Primary antibodies and reagents used in this study.**

| **REAGENT or RESOURCE** | **SOURCE** | **IDENTIFIER** |
| --- | --- | --- |
| **Antibodies** | | |
| Mouse anti-NEK2 | Proteintech | Cat#66632-1-Ig |
| Rabbit anti- NEK2 | Abcam | Cat# ab227958 |
| Mouse anti-FOXO1 | Proteintech | Cat#66457-1-Ig |
| Rabbit anti-FOXO1 | Proteintech | Cat#18592-1-AP |
| Mouse anti-HA | Sigma-Aldrich | Cat#H3663 |
| Mouse anti-Flag | Sigma-Aldrich | Cat#F1804 |
| Mouse anti-β-actin | Sigma-Aldrich | Cat#A1978 |
| Rabbit anti-HA | Proteintech | Cat#51064-2-AP |
| Rabbit anti-Flag | Proteintech | Cat#20543-1-AP |
| Rabbit anti-β-actin | Proteintech | Cat#20536-1-AP |
| Rabbit anti-Ubiquitin | Proteintech | Cat#10201-2-AP |
| IRDye 800CW goat anti-rabbit | LI-COR | Cat#926-32211 |
| IRDye 800CW goat anti- mouse | LI-COR | Cat#926-32210 |
| IRDye 680RD goat anti-mouse | LI-COR | Cat#926-68070 |
| IRDye 680RD goat anti-rabbit | LI-COR | Cat#926-68071 |
| Cycloheximide | MedChemExpress | Cat#HY-12320 |
| MG132 | MedChemExpress | Cat#HY-13259 |
| Lipofectamine 2000 | Thermo Fisher | Cat#11668019 |
| INH1 | MCE | HY-16660 |
| Mouse anti-phosphothreonine | Cell signaling | Cat#9386 |
| Rabbit anti-phosphoserine | Abcam | Cat#ab9332 |
